# Antifungal effects of ethanolic and aqueous extracts of *Vitex* *agnus-castus* against vaginal isolates of *Candida albicans*

**DOI:** 10.18502/cmm.4.1.26

**Published:** 2018-03

**Authors:** Nasser Keikha, Mahdieh Shafaghat, Seyed Mohamad Mousavia, Mahdiyeh Moudi, Farshid Keshavarzi

**Affiliations:** 1 Medical Laboratory Sciences Department, Infectious Diseases and Tropical Medicine Research Center, Resistant Tuberculosis Institute, Zahedan University of Medical Sciences, Zahedan, Iran; 2 Plant Biology Department, Infectious Diseases and Tropical Medicine Research Center, Resistant Tuberculosis Institute, Zahedan University of Medical Sciences, Zahedan, Iran; 3 Plant Biology Department, Zabol Medicinal Plant Research Center, Zabol University of Medical Sciences, Zabol, Iran; 4 Medical Genetics Department, Genetics of Non-Communicable Disease Researc Center, Zahedan University of Medical Sciences, Zahedan, Iran; 5 Clinical Biochemistry Department, Zahedan University of Medical Sciences, Zahedan, Iran

**Keywords:** Antifungal activity, *Candida albicans*, *Vitex agnus-castus*

## Abstract

**Background and Purpose:**

Vulvovaginal candidiasis is one of the most common infections in female genital organs, which is caused by *Candida* species. *Candida albicans* is the causative agent of more than 80% of infections, and the role of non-*Candida* strains in the disease etiology is less prominent. The expansion of Azoles resistance among *C. albicans* strains is considered an important medical problem. According to previous studies, *Vitex agnus-castus* (vitex) has some antimicrobial effects. We aimed to evaluate the anti-fungal effects of aqueous and alcoholic extracts of vitex against clinical vaginal isolates of *C. albicans* in comparison with fluconazole.

**Materials and Methods:**

Gas chromatography-mass spectrometry analysis was performed on vitex to identify its possible bioactive components. Forty *C. albicans* clinical isolates were identified by using germ tube, chlamydospore production, culture on CHROMagar, and polymerase chain reaction-restriction fragment length polymorphism (PCR-RFLP). Finally, after the extraction of vitex, drug susceptibility test was carried out according to the clinical laboratory standards institute (CLSI) M27-S4 document guidelines.

**Results:**

The major chemical components of vitex leaf as determined by gas chromatography included α-Pinene, isoterpinolene, caryophyllene, and azulene. The minimum inhibitory concentrations (MICs) of aqueous and alcoholic extracts of vitex, as well as fluconazole were within the ranges of 15.62–62.5, 7.81–15.62, and 0.25–8 μg/mL, respectively.

**Conclusion:**

Our findings showed that the alcoholic and aqueous extracts of vitex had antifungal activity against clinical isolates of *C. albicans*. Moreover, the alcoholic extract of vitex and fluconazole were more effective against clinical vaginal isolates of *C. albicans* compared to the aqueous extract of vitex.

## Introduction

In recent years, traditional medicine has been increasingly applied all over the world, which is due to the lower risk and cost of these medications. According to different studies, *Vitex agnus-castus *(vitex) is one of the most significant plants that has antibacterial effects and can treat diseases that have become resistant to chemical medications after a while [1]. Some researchers have recommended this herb for the treatment of acne due to its antifungal, anti-androgenic, and antibacterial properties. Moreover, this plant can be effective in treating hyperprolactinemia [2]. 

Vitex is native to the Middle East and Europe, and it is traditionally used to treat many diseases in women, including endometriosis, abnormal menstrual cycles, relief of menopausal symptoms, and insufficient breast milk [3, 4]. Inducing apoptosis in cancer cells, the polyphenolic compounds (e.g., flavonoids) derived from this plant act as anticancer agents. Furthermore, this plant can decrease blood cholesterol and prevent osteoporosis [5]. The extract of this herb was reported to have satisfactory effects on the learning and memory of rats, which is probably due to the increased expression of estrogen receptor alpha (*ERα*) genes [6]. 

Vitex consists of such chemical compounds as α-Pinene, cis-Ocimene, 1,8-cineole, β-Farnesene, Terpinen-4-ol, α-Terpineol, and caryophyllene. This herb is used for its carminative, antiseptic, and various biological activities such as antimicrobial and antioxidant purposes [7]. Vitex extract increases progesterone level and reduces estrogen level by affecting the level of follicle-stimulating and luteinizing hormones secreted in the pituitary gland. In addition, it can reduce the high level of prolactin before menstruation through dopaminergic mechanisms [8]. Therefore, with regard to the previous studies and drug resistance of the fungal infections caused by *Candida albicans*, it seems necessary to find a better alternative and develop more effective antifungal medications [9, 10]. 

Vulvovaginal candidiasis, which is a vaginal yeast infection caused by *Candida* species, is one of the most common causes of women’s referral to clinics. Almost 20% of women harbor *C. albicans* in the vagina without having any symptoms [11]. This fungus is a species that creates both yeast and mycelium at high pH levels and temperatures. *Candida albicans* is the normal flora of the digestive system and can be found in the adults’ oral cavity in limited numbers [12]. According to the literature, *C. albicans* accounts for 85–90% of vaginal yeast infections, followed by *C. tropicalis* and *C. glabrata*. These fungi, which are also called opportunistic fungi, are the most common causes of disease, especially in patients with immunodeficiency [13].

Given the resistance of *C. albicans* to the conventional antifungal medications and the lack of a proper treatment, traditional medicines and herbal extracts are still being used as therapeutic options for this purpose [14]. We aimed to evaluate the effects of aqueous and alcoholic extracts of vitex on the clinical vaginal isolates of *C. albicans* in comparison with fluconazole.

## Materials and Methods

***Sampling clinical isolates of*** ***Candida***

This cross-sectional non-randomized study was conducted on 450 patients suspected of vulvovaginal candidiasis visiting the specialized gynecology clinics of Zahedan, Iran. Sampling was carried out over a course of six months (August 2016–January 2017). The study protocol was approved by the Medical Research Ethics Committee of Zahedan University of Medical Sciences (IR.ZAUMS.REC.1396.390). After obtaining informed consents from the patients, two vaginal swabs were collected from each participant and sent to a laboratory. 

First, one of the swabs was cultured on Sabouraud Glucose Agar (SGA) containing chloramphenicol at 35°C for 48 h. The second swab was used for direct microscopic examination. In the next stage, we performed phenotypic tests, including mass production of germ tube, chlamydospores, and culture on CHROMagar *Candida* medium (Paris, France) to detect 40 clinical isolates of *C. albicans*. We used *C. albicans *standard clinical isolate of  ATCC 10231.

***Molecular identification***

***DNA extraction and PCR amplification***

The genotypic identification of yeast species was carried out for the confirmation of 40 clinical isolates of *C. albicans*. To this aim, DNA samples were extracted from *C. albicans* clinical isolates and standard strains using the phenol-chloroform method with the aid of a glass pearl and 48-hour fresh yeast cultures [15]. After the evaluation of the quality and quantity of the extracted DNAs, polymerase chain reaction (PCR) was performed using ITS1: TCCGTAGGTGAACCTGCG and ITS4: CCTCCGCTTATTGATATGC primers (Tag Copenhagen, Denmark) for the proliferation of ITS1-5.8S-ITS2 Rdna regions following the Master Mix kit protocol of Thermo Scientific. 

The substances used in the PCR, including Master Mix 12.5 µL, primer F 1.5 µL, primer R 1.5 µL (10 Picomol), template DNA 50 ng, and diethyl pyrocarbonat (DEPC)-treated Water up to 25 µL, were added to the microtube. We performed gradient PCR at temperatures ranging from 55°C to 60°C, and the optimal melting temperature of the primers was found to be 56°C. The PCR conditions were as follow: 30 cycles of denaturation at 94°C for 2 min, annealing at 56°C for 30 seconds, and extension at 72°C for 2 min.

***PCR-restriction Fragment Length Polymorphism reaction with MspI enzyme***

In this stage, *MspI* enzyme (Thermo Fisher Scientific, Lithuania) was used to differentiate between *C. albicans*, *C. glabrata*, *C. parapsilosis*, *C. tropicalis,* and *C. krusei *species. About 10 μl of the PCR product was combined with 2 μl of enzyme buffer, 1 μl of *MspI * enzyme, and 7 μl of sterilized distilled water, and then it was kept at 37°C for 2–3 h. Afterwards, the PCR products were analyzed by 2% agarose electrophoresis (*MspI * enzyme cuts the proliferated pieces at CCGG region). Eventually, *Mbo1* enzyme was applied for the differentiation of *C. albicans* from *C. dubliniensis*.

***Collection of Vitex*** ***agnus-castus***

After performing the field evaluations, vitex was collected from kilometer 10 of Zabol-Nehbandan road. The plant was identified by the Botany Herbarium of Agriculture Research Institute, Zabol University, Iran. Subsequently, the leaves were separated from the pedicles and dried in the shade. 

***Preparation of the alcoholic and aqueous extracts of Vitex*** ***agnus-castus***

After complete grinding of the leaves, specific amounts of ethanol and distilled water were added to them. The compound was slowly mixed on a rotator for 48 h. Subsequently, the solvent and herb mixture was separated using a filter paper to obtain the initial extract. In this study, 70% ethanol was applied using the percolation method. Accordingly, 50 g of vitex powder was poured into a decanter and added to 70% ethanol step by step. Ethanol was added after getting warmed and then placed in the decanter. This process was continued until all the powder inside the decanter got wet and a slight amount of ethanol was on the surface of the sample inside the decanter. We spent 24 h for the extraction process to let the plant powder fully absorb the solvent and facilitate the solution of the maximum amount of effective compound in ethanol. After this process, the solvent was separated from the extract using a centrifuge and vacuum pump. At the end, the obtained extracts were filtered by Millipore Syringe Filter with 0.22 µm pore size hydrophilic polyvinylidene difluoride (PVDF) membrane [16].

**2.2.5.** ***Gas chromatography and mass spectroscopy (GC-MS) analysis***

GC-MS analysis of vitex was performed on GC 6890 equipped with MS 5973-HO detector and HP-5MS capillary column (30 × 0.25 m, 0.25 µm; Agilent Co., USA) operating at 70 eV ionization energy. The initial column temperature was set at 60˚C, then it was increased from 60˚C to 220˚C (heating rate: 5ºC per minute), from 190˚C to 270˚C for 30 min, and finally kept at 270˚C for approximately 5 min; the total analysis time was about 34 min.

**2.2.6.** ***Vitex*** ***agnus-castus*** ***and fluconazole susceptibility test***

Fluconazole (Amin Chemical & Pharmaceutical Co, Iran), as well as alcoholic and aqueous extracts of vitex were prepared in 100% dimethyl sulfoxide. Alcoholic and aqueous extracts of vitex and fluconazole were diluted in 0.05% dimethyl sulfoxide and distilled water, respectively, to provide the following concentrations: 0.48–250 µg/mL for alcoholic and aqueous extracts of vitex and 0.06–64 µg/mL for fluconazole; all the solutions were stored at –20°C.

Drug susceptibility test was performed using the M27-S4 standard protocol presented by the Clinical and Laboratory Standards Institute (CLSI) [17].

 fresh cultures of 40 clinical vaginal isolates of *C. albicans,* approved by the phenotype and genotype methods, was prepared on SGA with chloramphenicol (European Division QUELAB, UK) after 48 h of incubation at 35°C. After the incubation and growth of *C. albicans* yeasts, the cell suspension of 0.5 x 10^3^ – 2.5 x 10^3^ CFU/ml was prepared. MIC was determined after incubation of the 96-well microplates for 24–48 h at 35°C. Based on the number of colonies grown in each concentration of alcoholic and aqueous extracts of vitex and fluconazole compared to the growth rate of the drug-free control well, the MIC90 and MIC50 of the fungus were obtained. 

According to CLSI M27-S4 (new CLSI breakpoint) interpretive breakpoints for *Candida *species and fluconazole, MIC ≤ 2 μg/ml was considered to show susceptibility, and MIC ≥ 8 μg/ml were considered to show resistance. All the tests were performed in triplicate.

***Statistical Analysis***

Differences among the mean values were determined by using Student’s t-test in SPSS, version 22.0. *P-value* less than 0.05 was considered statistically significant. 

**Results**

The results of PCR indicated that the size of DNA fragment was 535 bp. The PCR-RFLP findings showed that the function of MspI enzyme on PCR products in all the *C. albicans* samples led to the production of two specific bands with the lengths of 238 and 297 bp. Finally, 40 clinical isolates were confirmed as *C. albicans* and used for the following analyses. 

GC-MS analysis revealed the presence of 36 compounds (Table 1), the major compounds included α-Pinene, isoterpinolene, caryophyllene, and azulene.

**Table 1:** Chemical composition of vitex leaves determined by gas chromatography

**Constituents**	**Percentage (%)**
α-Pinene	15.2
Sabinene	2.1
2-β-pinene	0.22
β -myrcene	0.81
Benzene	0.34
Dl–Limonene	5.8
γ-Terpinene	0.39
α-Terpinolene	0.41
Isoterpinolene	19.90
Cis-2,6,7-Dimethyl-2,6-octadiene	0.63
Caryophyllene	18.7
Bicyclo(3-1-1)Hept-2-ene	0.29
α-humulene	1.61
H-cycloproazulene	0.33
β-selinene	0.47
α-Seliene	0.27
Cyclohexen	0.35
1,4-methanoazulene	2.39
Azulene	12.8
Aromadenderne	0.39
3-methylene-bicycle	0.18
1H-Inden-1-ethylideneoctahydro-7a	1.65
Naphtalene, hexahydro-1,6dimethyl	0.72
γ -himachalene	0.42
Nerolidol	0.15
3-(trifluoromethyl)5-penthyl	0.10
Phenol-2,4-bis(1,1dimethyethyl )	7.98
Cheloviolene	1.62
1H-Naphto (2,1-b)pyran	0.61
5-Amino-6-nitroquinoline	0.29
3,7,11,15 tetramethyle hexade	2.34
17,17-D2–Androst-5-en	3.21
Cembrene	1.15
Tetra cyclo	0.33
Sigmoscepter lin-A	1.15
Exo–avarone	0.24

**Table 2:** In vitro antifungal susceptibilities of aqueous and alcoholic extracts of *Vitex agnus-castus *and fluconazole against the clinical isolates of  C. albicans*

**Isolate**	**Antifungal agent**	**MIC** **range (μg/mL)**	**MIC90 (μg/mL)**	**MIC50 (μg/mL)**	**Resistant (Number)**
Forty clinical vaginal isolates of *C. albicans*	Aqueous extract of vitex	15.62–62.5	62.5	31.25	ND
	Alcoholic extract of vitex	7.81–15.62	15.62	7.81	ND
	Fluconazole	0.25–8	1	0.25	8

MIC: minimum inhibitory concentration

ND: Non defined

Note: * Values are expressed as means of three independent experiments (*P* < 0.05).

The MICs of aqueous and alcoholic extracts of vitex against the clinical isolates of *C. albicans* were 15.62–62.5 and 7.81–15.62 μg/mL, respectively. On the other hand, the MICs of fluconazole for the evaluated clinical isolates were within the range of 0.25–8 μg/mL. Table 2 presents the results obtained from evaluating the antifungal effects of aqueous and alcoholic extracts of vitex and fluconazole against the clinical vaginal isolates of *C. albicans*.

## Discussion

Regarding the resistance of *C. albicans* to the conventional antifungal agents and considering the positive attitude of people toward herbal medicines, the search for other medications with higher effectiveness and less toxicity has attracted wide attention [10, 14, 18]. In previous studies, vitex was reported to have antibacterial activity. In a study conducted by Afarin et al., it was revealed that the MICs of the essential oil of aerial parts of vitex were 112.5 and 56.25 μg/mL against *C. albicans* PTCC 5027 and *Staphylococcus aureus* ATCC 6538, respectively [5]. Furthermore, Katiraei et al. evaluated the antifungal effect of vitex on some *Candida* species. In the mentioned study, the MIC of the extract against *C. albicans*, *C. tropicalis*, *C. parapsilosis,* and *C. dubliniensis* was 3.1 μg/mL; furthermore, against *C. krusei*, the MIC of vitex extract was reported to be 12.5 μg/mL [3].

In another study, Yilar et al. investigated the antifungal effects of vitex extract on some fungi. The tests revealed that this extract had a high range of antifungal effects, such that 5 µl of vitex essential oil reduced 90.23%, 55.68% and 63.35% of the mycelial growth in *Verticillium dahlia*, *Sclerotinia sclerotiorum,* and *Rhizoctonia solani*, respectively [19]. Asdadi et al. evaluated the antifungal effect of vertex seed extract on four *Candida* species isolated from cases of nosocomial infection. According to their results, minimum fungicidal concentration was 7 μg/mL for *C. albicans, C. glabrata, C. krusei *and* C. dubliniensis* [20]. 

In a study carried out by Ghannadi et al., the antibacterial activity of vitex extract was assessed using the disk-fusion method. In the mentioned study, the highest inhibitory effect was against *Staphylococcus aureus* with the growth inhibition zone of 50 mm [21]. According to the results of the present study, the alcoholic and aqueous extracts of vitex inhibited the growth rate of 50% of the clinically isolated *C. albicans* at the concentrations of 31.25 and 62.5 ml, respectively. Moreover, the MIC90 of the alcoholic and aqueous extracts of vitex were 15.62 and 31.25 µg/mL, respectively.

In contrast with the results reported by previous studies, in the current study, fluconazole and alcoholic extract of vitex showed better activity against clinical vaginal isolates of *C. albicans*, compared to the aqueous extract of vitex. According to our results, the alcoholic extract of vitex had more powerful effects against clinical vaginal isolates of *C. albicans* compared to the aqueous extract of this plant.

In the present study, 20% of the *C. albicans* isolates (n=8) were resistant to fluconazole. Comparing the efficacy of fluconazole against *C. albicans*, previous studies reported inconsistent results. In the studies by Shokohi et al. and Gross et al., resistance of *C. albicans* isolates to fluconazole was estimated at 2.6% and 3.5%, respectively [22, 23]. 

Our results showed that the alcoholic extract of vitex had MIC range lower than those of fluconazole. Thus, the alcoholic extract of vitex can be considered a more functional antifungal drug than fluconazole. After performing complementary evaluations, the application of alcoholic and aqueous extracts of vitex is recommended for the control of infections caused by *C. albicans* regarding resistance to fluconazole and the positive attitude of people toward herbal medicines.

## Conclusion

According to the results of the current study, the alcoholic and aqueous extracts of vitex had antifungal activity against the clinical vaginal isolates of *C. albicans*. Moreover, alcoholic extract of vitex followed by fluconazole and aqueous extract of vitex had the highest activity against clinical vaginal isolates of *C. albicans*, respectively.
